# Functional Efficacies of Humate and β-Mannanase Against Aflatoxin B1 and Deoxynivalenol in the Diets for Nursery Pigs

**DOI:** 10.3390/toxins17090456

**Published:** 2025-09-11

**Authors:** Yesid R. Garavito-Duarte, Jeonghyeon Son, Alexandra C. Weaver, Sung Woo Kim

**Affiliations:** Department of Animal Science, North Carolina State University, Raleigh, NC 27695, USA; yrgaravi@ncsu.edu (Y.R.G.-D.); json7@ncsu.edu (J.S.); aweaver@alltech.com (A.C.W.)

**Keywords:** aflatoxin, deoxynivalenol, humate, β-mannan hydrolysate, β-mannanase, mycotoxins, nursery pigs

## Abstract

After in vitro mycotoxin binding validation, humate and β-mannanase were tested for mitigating the negative effects of aflatoxin B_1_ and deoxynivalenol. Gilts at 8.7 ± 0.5 kg body weight were allotted to four treatments: NC (uncontaminated diet); PC (contaminated diet: 150 µg aflatoxin B_1_ and 1100 µg deoxynivalenol per kg feed); HT (PC + humate, 0.5%); and EM (PC + β-mannanase, 800 U/kg diet). Growth performance was recorded for 42 days, and blood and tissue samples were collected for hematological and histopathological evaluations. The PC reduced (*p* < 0.05) serum tumor necrosis factor-α at day 28 and tended to increase (*p* = 0.062) immunoglobulin G (IgG), whereas the EM reduced IgG (*p* < 0.05) at day 42. The PC increased (*p* < 0.05) mean corpuscular hemoglobin and volume, which were reduced (*p* < 0.05) by HT or EM at day 42. The PC increased (*p* < 0.05) bile duct hyperplasia, which was attenuated (*p* < 0.05) by HT or EM. The PC reduced (*p* < 0.05) gain- to-feed ratio for the overall period, whereas HT increased (*p* < 0.05) average daily gain on days 21 to 28. These results suggest that HT and EM may mitigate mycotoxin-induced immune and hepatic damage in pigs through adsorbing mycotoxins.

## 1. Introduction

Mycotoxins are toxic secondary metabolites produced by fungi that frequently contaminate feedstuffs and pose serious health and productivity risks to pigs [1]. The severity and nature of mycotoxin-induced toxicity are influenced by multiple factors, including the type and dose of the mycotoxin, environmental conditions, and the physiological status of the animal [2]. Among the most common and deleterious mycotoxins are aflatoxins, fumonisins, and deoxynivalenol (DON) [3,4], which can negatively affect intestinal microbiota, induce immune responses and oxidative stress, disrupt intestinal morphology, impair liver and kidney function, and impair nutrient digestion in the small intestine, leading to reduced growth performance [5,6]. Due to the widespread presence of mycotoxins in cereal grains used in pig diets, mitigation strategies are essential to maintain pig health and growth performance. One common approach is the use of mycotoxin-detoxifying agents. These agents act through different mechanisms, such as adsorption of mycotoxins in the gastrointestinal tract [2], enzymatic or microbial degradation into non-toxic metabolites [2,7], and enhancement of immune function and intestinal integrity to reduce susceptibility to mycotoxins [7]. Common mycotoxin-detoxifying agents include inorganic binders such as clays (e.g., bentonite, zeolite) and other minerals; organic binders such as yeast cell wall derivatives, which can adsorb mycotoxins aflatoxin B_1_ (AF) and DON [8,9]; and mycotoxin-biotransforming agents, including mycotoxin-degrading enzymes and specific bacterial strains that degrade mycotoxins into non-toxic metabolites [2,7,10]. In addition, functional feed additives such as prebiotics, probiotics, postbiotics, phytobiotics, and synbiotics, are used to enhance intestinal health and immune responses, thereby improving the animal’s ability to cope with systemic effects of mycotoxins [11].

Humate is mined humic substances including humic acid (10 to 55%), fulvic acid (1 to 25%), and humin, as well as trace minerals including iron, manganese, and copper [12]. Humic acids and fulvic acids are high-molecular-weight organic acids that have been reported to improve growth performance, enhance loin color, and reduce ammonia emissions from manure when included in pig diets at 0.5 to 1.0% [13,14]. The functional groups in humate provide ion-exchange capacity, which may allow adsorption of mycotoxins in the gastrointestinal tract [15,16,17]. Previous studies have shown that humate can alleviate the negative effects of aflatoxin on broiler growth performance by adsorbing aflatoxins [17,18].

Commercial pig diets contain 0.5 to 1.0% of β-mannans and β-galactomannans, which are contributed by protein supplements including soybean meal, palm kernel meal, and corn distillers dried grains with solubles [19]. β-mannans and β-galactomannans possess anti-nutritional characteristics impairing intestinal health [20,21], reducing nutrient digestibility [21], and thus reducing growth efficiency of nursery pigs [21]. To counter these effects, supplementation with β-mannanase is commonly used in pig production. In addition to reducing the anti-nutritional effects of β-mannans and β-galactomannans, β-mannanase hydrolysis produces mannan-oligosaccharides (MOSs). These MOSs may also provide functional benefits; recent studies indicate their physicochemical ability to bind and inactivate aflatoxins [22,23,24].

Evaluation of the mycotoxin-binding capacity of humate and β-mannan hydrolysate provides innovative and practical implications for handling mycotoxins in pig production. Humate, rich in humic and fulvic acids, and β-mannan hydrolysate, generated through in-feed β-mannanase supplementation, possess distinct physicochemical properties that may reduce the negative impacts of dietary AF and DON. It is hypothesized that dietary inclusion of humate (0.5%) and β-mannanase (800 U/kg feed) to hydrolyze β-mannans would provide protective effects against AF and DON contamination by modulating systemic inflammation, preserving organ integrity, and supporting growth performance in nursery pigs.

## 2. Results

### 2.1. In Vitro Mycotoxin Binding Efficacy of Humate and β-Mannan Hydrolysate

Among the evaluated alternatives, humate achieved 28.5% aflatoxin adsorption and 6.1% desorption, resulting in a net binding efficiency of 22.4% (Table 1), whereas β-mannan hydrolysate exhibited 22.2% adsorption and 5.6% desorption, corresponding to a 16.6% binding efficiency. In comparison, a more conventionally available clay-based mycotoxin-detoxifying additive showed the highest binding capacity with 92.0% adsorption and 0.8% desorption, resulting in a net efficiency of 91.2%, followed by a clay and yeast cell wall-based mycotoxin-detoxifying additive, which achieved 75.2% adsorption and 5.7% desorption, yielding a net binding efficiency of 69.5%.

### 2.2. In Vivo Evaluation of Humate and β-Mannanase in Nursery Pig Diets Naturally Contaminated with Aflatoxins and Deoxynivalenol

#### 2.2.1. Immune Response

At day 28, pigs in the positive control (PC) treatment had lower (*p* < 0.05) serum tumor necrosis factor α (TNF-α) concentrations compared with pigs in the negative control (NC) treatment (Table 2). However, there were no differences in TNF-α concentrations between pigs in the PC and those either in the HT treatment (PC with mined humate at 0.5%) or EM treatment (PC with β-mannanase at 800 U/kg to generate β-mannan hydrolysate). Serum immunoglobulin G (IgG) concentrations at day 28 did not differ among treatments groups.

At day 42, serum TNF-α concentrations remained unaffected between treatments groups (Table 2). Pigs in the PC treatment tended to have higher (*p* = 0.062) serum IgG compared with pigs in the NC treatment. However, pigs in the HT treatment tended to have reduced (*p* = 0.090) serum IgG levels compared with pigs in the PC treatment, whereas pigs in the EM treatment had lower (*p* < 0.05) serum IgG concentrations compared with those in the PC treatment.

#### 2.2.2. Hematological Profiles

At day 28, no differences were observed in hematocrit or hemoglobin concentrations among treatments groups (Table 3). Pigs in the PC treatment group had higher (*p* < 0.05) mean corpuscular hemoglobin (MCH) compared with pigs in the NC treatment group. However, pigs in the EM treatment group had lower (*p* < 0.05) MCH than those in the PC treatment group, whereas pigs in the HT treatment group tended to have reduced (*p* = 0.093) MCH compared with pigs in the PC treatment group. No effects on mean corpuscular hemoglobin concentration (MCHC) were observed in pigs in the PC treatment compared with those in the NC treatment. However, pigs in the EM treatment tended to have reduced (*p* = 0.078) MCHC compared with pigs in the PC treatment. No difference was observed between pigs in the PC treatment and those in the HT treatment. Mean corpuscular volume (MCV) was higher (*p* < 0.05) in pigs on the PC diet compared with pigs in the NC treatment, but no differences were found between the PC and either the HT or EM treatments. No differences were observed among treatments groups for platelet count, red blood cell (RBC) count, or white blood cell (WBC) count. Basophil counts were lower (*p* < 0.05) in pigs on the PC diet compared with those in the NC treatment, but no differences were observed between the PC and either the HT or EM treatments. Eosinophil, lymphocyte, monocyte, and neutrophil counts were not different among treatments groups.

At day 42, hematocrit levels tended to be higher (*p* = 0.080) in pigs on the PC diet compared with those fed the NC diet (Table 4). No differences in hematocrit were observed between the pigs in PC diet and those fed either the HT or EM treatment. Hemoglobin concentrations were not different among any of the treatment groups. Pigs in PC treatment group had higher (*p* < 0.05) MCH compared with pigs in the NC treatment. However, pigs in the EM treatment group had lower (*p* < 0.05) MCH than those in PC treatment, with no differences between pigs in the PC and HT treatments. Mean corpuscular hemoglobin concentration did not differ between pigs in the NC and PC treatment groups. Pigs in the EM group tended to have lower (*p* = 0.068) MCHC compared with those in the PC treatment group, whereas pigs in HT had higher (*p* < 0.05) MCHC compared with pigs in the PC treatment group. Mean corpuscular volume increased (*p* < 0.05) in pigs in the PC treatment compared with those in the NC treatment. Pigs in either the HT or EM treatment had lower (*p* < 0.05) MCV values compared with pigs in the PC treatment. No differences were observed between pigs in the NC and PC treatment for RBC count; however, RBC tended to increase (*p* = 0.052) in pigs in the EM treatment compared with those in PC treatment. Basophil counts were higher (*p* < 0.05) in pigs in the PC treatment compared with pigs in NC treatment, with no difference observed between pigs in either the HT or EM treatment. No differences were observed among treatments groups for platelet count or WBC count. Eosinophil counts tended to be lower (*p* = 0.052) in pigs in the EM treatment compared with pigs in the PC treatment. No differences were found between pigs in the NC and PC treatments, or between pigs in the PC and HT treatments. Monocyte counts tended to be higher (*p* = 0.074) in pigs in the PC treatment compared with pigs in the NC treatment and were lower (*p* < 0.05) in pigs in the EM treatment compared with pigs in the PC treatment. Pigs in the HT treatment tended to have reduced (*p* = 0.059) lymphocyte count compared with pigs in the PC treatment. No treatment effects were observed for neutrophil counts.

#### 2.2.3. Serum Biochemistry

At day 28, no differences were observed in serum glucose, total protein, blood urea nitrogen (BUN), globulin (GLOB), albumin-to-globulin ratio (ALB:GLOB), aspartate aminotransferase (AST), alanine aminotransferase (ALT), cholesterol, sodium, potassium, chloride, or calcium concentrations among treatment groups (Table 5). Serum creatinine (CRT) tended to be lower (*p* = 0.053) in pigs in the PC treatment compared to those in the NC treatment. Pigs in the EM treatment had higher (*p* < 0.05) CRT concentrations than pigs in PC treatment. The BUN-to-creatinine ratio (BUN:CRT) was higher (*p* < 0.05) in pigs in the PC treatment compared with pigs in NC treatment, with no differences between pigs in the PC treatment and either the HT or EM treatments. There were no differences in albumin (ALB) in pigs in the PC treatment compared with those in NC treatment, or between pigs in the PC and EM treatments, whereas pigs in the HT treatment tended to have higher (*p* = 0.079) serum ALB compared to pigs in PC treatment. Additionally, the sodium-to-potassium ratio (Na:K) tended to decrease (*p* = 0.075) in pigs in the EM treatment compared to those in PC treatment. Alkaline phosphatase levels were not different between pigs in the NC and PC treatment; however, pigs in the HT treatment tended to have lower (*p* = 0.093) alkaline phosphatase concentrations compared with pigs in PC treatment. Serum creatine phosphokinase (CPK) tended to decrease (*p* = 0.067) in pigs in the PC treatment compared to those in NC treatment. Bilirubin concentrations did not differ among treatments groups.

At day 42, no differences were observed in serum glucose, total protein, BUN, CRT, BUN:CRT ratio, ALB, GLOB, ALB:GLOB ratio, cholesterol, sodium, potassium, Na:K ratio, chloride, phosphorus, alkaline phosphate, and CPK concentrations among treatment groups (Table 6). Pigs in the PC treatment had higher (*p* < 0.05) AST concentrations than pigs in NC treatment. Pigs in the PC treatment tended to have lower (*p* = 0.073) serum ALT concentration compared to those in HT treatment. The serum calcium concentration was higher (*p* < 0.05) in pigs in the PC treatment compared with pigs in NC treatment.

#### 2.2.4. Organ Weight

No differences were observed among treatments for liver and kidney weights (Table 7). Pigs in the PC treatment had lower (*p* < 0.05) spleen weight compared to pigs in NC treatment. However, no differences were observed between pigs in the PC treatment and those in either the HT or EM treatments. Relative liver weight (as a percentage of body weight) was higher (*p* < 0.05) in pigs in the PC treatment compared to pigs in NC treatment. Although relative liver weight was not different between pigs in the PC treatment and pigs in EM treatment, it tended to be lower (*p* = 0.052) in pigs in the HT treatment compared to those in PC treatment. Relative kidney and spleen weights were not different among treatments. No differences were observed in liver, kidney, or spleen color parameters (lightness, redness, and yellowness) among treatments. Spleen lightness tended to be higher (*p* = 0.072) in pigs in the PC treatment compared to those in NC treatment, but was unaffected on pigs in the HT or EM treatments. Spleen redness increased (*p* < 0.05) in pigs in the PC treatment compared to pigs in NC treatment. No differences were observed between treatments groups for spleen yellowness.

#### 2.2.5. Tissue Damage

At the histological level, pigs in the PC treatment showed greater (*p* = 0.019) bile ductule hyperplasia in the liver compared to pigs in NC treatment (Table 8). Pigs in the HT or EM treatments had a reduced (*p* < 0.05) bile duct hyperplasia compared to pigs in PC treatment. Conversely, hepatic fibrosis was lower (*p* < 0.05) in pigs in the PC treatment than pigs in NC treatment; however, fibrosis did not differ between pigs in the PC treatment and those in HT or EM treatments. No differences were observed in hydropic degeneration between pigs in the PC and NC treatments, whereas pigs in HT treatment tended to show lower hydropic degeneration compared with pigs in PC treatment. Inflammation and necrosis in the liver were not different among treatments groups. Hepatic karyomegaly was increased (*p* < 0.05) in pigs in the PC treatment compared to pigs in NC treatment. No differences were observed between pigs in the PC treatment and either the HT or EM treatments. Liver vacuolation tended to be greater (*p* = 0.089) in pigs in the PC treatment compared to those in NC treatment. Pigs in the HT treatment tended to show lower (*p* = 0.089) liver vacuolation compared to those in PC treatment, whereas no differences were observed between pigs in the PC treatment and those in EM treatment. No differences were observed among treatments groups for the kidney for fibrosis, necrosis, protein casts, regeneration, or vacuolation.

#### 2.2.6. Growth Performance

Body weight was no different among treatments at any time points (days 7, 14, 21, 28, 35, or 42; Table 9). Average daily gain (ADG) did not differ among treatments groups from days 0 to 21. From days 21 to 28, pigs in the HT treatment showed greater (*p* < 0.05) ADG compared to pigs in PC treatment. No differences in ADG were observed between pigs in the PC treatment and those in EM treatment during any period. Overall ADG (day 0 to 42) was not different among treatments. Average daily feed intake (ADFI) was not affected by treatments during any period. The gain-to-feed ratio (G:F) from days 0 to 7 was lower (*p* < 0.05) in pigs in the HT treatment compared with pigs in PC treatment. From days 21 to 28, pigs in the HT treatment tended to have a higher (*p* = 0.077) G:F ratio than pigs in PC treatment. From days 28 to 35, pigs in the PC treatment tended to have a lower (*p* = 0.066) G:F ratio than those in NC treatment. In the overall period, pigs in the PC treatment had a lower (*p* < 0.05) G:F ratio compared to pigs in NC treatment, with no differences between pigs in the PC treatment and those in either the HT or EM treatments.

## 3. Discussion

This study provides insights into how humate and β-mannanase supplementation may mitigate some of the physiological and immunological disturbances induced by dietary mycotoxin exposure in nursery pigs. Although performance parameters were not directly affected, several systemic and histological markers highlighted the negative effects of mycotoxins and the potential for dietary intervention.

As a preliminary test, the AF binding capacities of humate, β-mannan hydrolysate obtained from guar gum, clay-based additive, and a clay and yeast cell wall-based additive were evaluated. Guar gum contains approximately 40% of β-mannans, which is more than 20 times higher than that in typically found in the commercial pig diets [19]. Due to this reason, a relatively high concentration of β-mannanase was used to mimic the in vivo dietary inclusion rate. Although humate and β-mannan hydrolysate exhibited less binding capacity compared with clay-based and clay and yeast cell wall-based additives, their binding capacities were similar to each other. This observation indicates that humate and β-mannan hydrolysate possess some aflatoxin binding capacity, but their efficacy remains limited compared with conventional binders. However, humate and β-mannan hydrolysate can be available at greater amount in feeds compared to additives and also provide other benefits beyond toxin binding, such as immunomodulation [22] and intestinal health support [25]. In vitro assays with DON were also attempted; however, reliable results could not be obtained because the high viscosity of guar gum and the large sample volume required for the assay clogged the HPLC column. Therefore, DON data are not reported in this study.

Regarding inflammation, mycotoxin exposure decreased serum TNF-α concentrations in pigs. This observation is consistent with previous research [26], which reported reduced TNF-α gene expression after aflatoxin exposure. Aflatoxins are commonly associated with reduced immunocompetence [27], potentially increasing susceptibility to secondary infection.

A possible mechanism by which mycotoxins impair immune responsiveness is the disruption of antigen-presenting cell function. Aflatoxins have been shown to compromise these cells, thereby reducing antigen–host interactions [28]. Because antigen presentation is a critical step in immune activation [29], its impairment can diminish downstream immune responses [30], including alterations in lymphocyte populations [31] and reductions in humoral immunity [32]. These mechanisms may partly explain the variability reported across studies. However, the biological significance of reduced TNF-α under chronic mycotoxin exposure remains complex and inconsistent [33,34,35], likely reflecting differences in basal diet composition or mycotoxin exposure levels.

Although IgG levels did not differ at day 28, the tendency for higher IgG in PC treatment at day 42 suggests a delayed or dysregulated humoral immune response. This increase may reflect persistent immune activation or altered antigen presentation caused by tissue damage [36,37]. Interestingly, both the HT and EM treatments mitigated these negative impacts, with EM showing the strongest reduction in IgG. This finding aligns with previous studies [33,38], suggesting that EM reduced IgG levels not only through mycotoxin adsorption but also via other mechanisms. Similarly, β-mannanase supplementation has been shown to linearly reduce serum IgG levels in nursery pigs as β-mannans can stimulate the immune system because immune cells recognize them as pathogen-associated molecular patterns, leading to unnecessary immune response such as IgG secretion [39]. However, β-mannanase-mediated hydrolysis of β-mannan is recognized by Toll-like receptors and cannot induce an immune response [18,23,40,41].

In the hematological profile, mycotoxin contamination increased both MCH and MCV values, which are directly associated with the oxygen-carrying capacity and iron status of pigs [42]. These findings suggest that the absorbed mycotoxins negatively affected the liver and spleen, thereby impairing RBC production and clearance, or disrupting iron metabolism [43,44]. The adverse impacts of mycotoxins on MCH and MCV were reduced by supplementation of HT and EM. Based on these findings, HT and EM may help relieve the negative impacts of mycotoxins on hematological parameters by adsorbing mycotoxins and preventing their absorption into the animal.

To evaluate the impacts of mycotoxin contamination on the liver and kidney function, serum biochemistry parameters were analyzed. On day 28, CRT decreased in the PC treatment. Creatinine is a metabolic byproduct of creatine, synthesized in the liver and excreted by the kidneys [11]. Mycotoxins absorption may have impaired both organs, indirectly affecting CRT production and excretion [45]. Consequently, BUN:CRT ratio increased due to the lower CRT concentration. Although humate and β-mannan hydrolysate showed similar mycotoxin binding capacities, EM treatment alleviated the negative impacts on CRT, whereas HT treatment did not change the serum CRT levels. The mechanisms are unclear, but absorbed MOSs may have contributed to liver and kidney recovery in addition to binding mycotoxins.

Serum phosphorus concentrations were elevated on both days 28 and 42 in response to mycotoxins. The kidneys regulate blood mineral concentrations through excretion [46], and damage from mycotoxins may impair this function, leading to increase serum phosphorus. Similarly, serum calcium levels were elevated, likely reflecting impaired renal functions. On day 42, serum AST concentration of pigs was increased by mycotoxins contamination. Elevated AST is a common symptom of mycotoxicosis [27]. Previous studies have shown that dietary AF and fumonisin increase serum AST [47,48]. As ingested mycotoxins are detoxified in the liver, hepatic cell damage can release intracellular enzymes, including AST, into the blood stream [49]. These results suggest that liver tissue was damaged by mycotoxins, and this damage was not alleviated by either HT or EM treatment.

In this study, mycotoxins contamination increased the relative liver weight in pigs. This is consistent with results reported from a previous meta-analysis [50]. The increase in liver weight is likely because absorbed mycotoxins are first transported to the liver for detoxification [51]. In contrast, the relative weight of the kidney and spleen were not affected, likely because of the liver’s role as the primary target organ. Although absolute spleen weight was reduced, this change could be related to an atrophy generated by mycotoxins. For example, DON can increase mitochondrial reactive oxygen species production, leading to spleen damage [52].

To evaluate the hepatic and renal toxicity of mycotoxins and potential recovery effects of humate and β-mannan hydrolysate, tissue damage was evaluated at histological level. Consistent with the changes observed in organ weight, mycotoxins negatively affected the liver but not the kidney. This discrepancy is likely due to the level of mycotoxin exposure, as the liver is the first organ to encounter absorbed mycotoxins, whereas the kidney is exposed only to the fraction of mycotoxins that have not been detoxified by the liver [53]. Among liver damage criteria, karyomegaly was notably increased by mycotoxins, consistent with previous studies in pigs and rats [33,54]. Karyomegaly is caused by chemicals or toxins that interfere with mitosis, causing abnormal nuclear enlargement due to the failed cell division [55]. Moreover, in a previous study Weaver et al. [33] observed that clay and clay with yeast alleviated karyomegaly. However, in this study neither humate nor β-mannan hydrolysate alleviated this condition. This is likely due to their limited mycotoxin binding capacity, which allowed enough absorption of mycotoxins to persistently induce karyomegaly.

Negative impacts of mycotoxins were also observed in blood profiles, serum biochemistry, organ weights, and histopathology. Despite these physiological effects, growth performance changes were limited and only detected between days 28 and 35. This suggests that the mycotoxin doses used in this study were insufficient to severely impair growth performance of pigs. Deoxynivalenol is known to reduce feed intake, as reported in a previous study by Holanda and Kim [56], and contamination with DON at 1900 µg/kg reduced ADG only during days 28 to 35. Similarly, Weaver et al. [57] reported that diets contaminated with DON (4800 µg/kg) and zearalenone (300 µg/kg) reduced feed intake by 38%, consequently impairing the growth performance of nursery pigs after 42 days of feeding. Variability in growth performance outcomes across studies may result from differences in mycotoxins combinations, dosages, and exposure durations [7]. At high, acute levels mycotoxins such as DON can induce vomiting and suppress feed intake [58]. Chronic exposure to lower concentrations could primarily reduce feed intake through mechanisms involving impaired intestinal motility [6], serotonin-mediated signaling [59], and inflammatory cytokines production [36]. Additionally, low levels of mycotoxins in the NC diet may have slightly impaired growth performance, reducing the contrast expected between PC and NC treatments [11,56,60]. Regarding feed additives, β-mannan hydrolysate did not improve growth performance of pigs, whereas humate improved ADG and tended to increase the G:F ratio during days 21 to 28. This observation is likely attributed to the protective role of humate in reducing organ damage, as evidenced by improved hematological and biochemical markers, which could enhance nutrient utilization or reduce the nutritional demands for tissue repair.

This study was designed to evaluate the negative impacts of AF and DON in nursery pigs and to determine the protective effects of HT and EM. Parameters related to detoxification, including liver function, tissue damage, and organ weight, were assessed. Although only gilts were used, these outcomes are unlikely to be influenced by sex, as AF and DON are not directly associated with sexual maturity or reproductive physiology. Nevertheless, larger and sex-balanced studies are warranted to confirm and extend the present findings.

The results demonstrate that although aflatoxins (150 µg/kg) and deoxynivalenol (1100 µg/kg) did not severely depress growth performance of pigs, they clearly disrupted immune function, hematological parameters, and liver function. Both humate and β-mannan hydrolysate offered protective functions against mycotoxins, but their mechanisms and efficacy differ. Humate appeared more effective at mitigating tissue damage and had period-specific improvements in ADG and the G:F ratio, whereas β-mannan hydrolysate exerted immune modulation and the stabilization of hematological profiles.

## 4. Materials and Methods

### 4.1. Humate, β-Mannan Hydrolysate, and Commerically Available Mycotoxin-Detoxifying Additives

The humate used in this study was a mined humic substance by Live Earth Product (Emery, UT, USA) containing humic acid (46.4%), fulvic acid (9.1%), crude protein (5.3%), iron (1.4%), and the remaining majority is humin.

Guar gum (MP Biomedicals, LLC, Solon, OH, USA), a mannan-rich substrate (40% β-mannan), was used to generate β-mannan hydrolysate by β-mannanase. For each replicate (n = 3), 10 g of guar gum was combined with β-mannanase (16,000 U/kg, CTCBIO, Seoul, Republic of Korea) and mixed with 500 mL of distilled water. The inclusion levels of β-mannanase (U/kg feed) were based on previous studies considering the amount of substrate per U [50,51]. One unit of β-mannanase activity was the amount of enzyme required to release 1 µmole of mannose reducing sugars equivalents per minute from 1.0% locust bean gum in 200 mM sodium phosphate buffer, pH 6.0 at 50 °C [50,51]. The mixtures were incubated in a shaking water bath at 30 °C and pH 7 for 5 h to facilitate enzymatic digestion. After incubation, samples were frozen at −20 °C, freeze-dried (VirTis 24D x 48, Scientific Products, Warminster, PA, USA), and ground for subsequent in vitro mycotoxin binding efficacy test.

Two commercially available mycotoxin-detoxifying additives were used. Clay-based additives (Calibrin^®^-A Enterosorbent) were composed of a processed calcium montmorillonite clay and obtained from a commercial company (Amlan International, Chicago, IL, USA). Clay and yeast cell wall-based additive (Unike, Nutriad International NV, Sint-Niklaas, Belgium) was composed of sodium bentonite, sepiolite clay, and brewer’s dried yeast.

### 4.2. In Vitro Mycotoxin Binding Efficacy Test

An in vitro assay was conducted to evaluate the AF binding efficiency of humate, β-mannan hydrolysate (released from guar gum), and two commercial clay-based binders (clay and clay with yeast). Each sample was tested at an inclusion rate of 3.0 mg/mL against a 3.0 µg/mL of AF. The in vitro assay binding procedures consisted of 2 phases to mimic the conditions of the stomach and the small intestine; an absorption phase conducted at pH 3.0, representing the acidic environment of the stomach, and a desorption at pH 6.5, reflecting the pH of the small intestine.

During the absorption phase, the interaction between the test compound and AF was allowed to proceed under controlled conditions. Following this, samples were adjusted to the desorption pH (6.5) to determine the stability of the binding under intestinal conditions. The percentage of mycotoxin bound during the absorption phase and released during the desorption phase was quantified. Binding efficiency was calculated as the difference between the absorption and desorption percentages, providing an estimate of the net capacity of each sample to stably bind AF under simulated digestive conditions. The concentration of AF was determined to calculate the mycotoxin absorption and desorption and binding efficiency using high-performance liquid chromatography (Agilent 1100 Series, Agilent Technologies, Santa Clara, CA, USA).

### 4.3. Animals, Design and Diets for the In Vivo Evaluation of Humate and β-Mannanase

The protocol for the use of animals was approved by the North Carolina State University Animal Care and Use Committee (Raleigh, NC, USA). The experiment was carried out at the North Carolina Swine Evaluation Station (Clayton, NC, USA).

Corn naturally contaminated with AF (270 µg/kg), originating from field contamination and confirmed by analysis using an Agilent 1100 Series HPLC system (limit of quantification: 20 µg/kg), and barley naturally contaminated with DON (30,000 µg/kg), quantified by an Agilent 1100 Series HPLC system (limit of quantification: 500 µg/kg), were used as the mycotoxins sources and no additional purified mycotoxins were added [33]. These grains were used to make a positive control diet, which contained target concentrations of 150 µg AF/kg and 1100 µg DON/kg (Table 10). Humate (0.5%, Live Earth Product, Emery, UT, USA) was added to the positive control diet. β-mannanase (800 U/kg feed) was added to positive control diet to hydrolyze β-mannans. The inclusion levels of β-mannanase (U/kg feed) were based on previous studies considering substrate availability per unit of enzyme [50,51]. The analyzed activity of β-mannanase in the EM diet was 986 U/kg feed. The NC diet was formulated using non-contaminated corn and barley, without purified mycotoxins.

A total of 96 gilts (initial body weight = 8.8 ± 0.4 kg; crossbred pigs; Smithfield Premium Genetics, Rose Hill, NC, USA) were used in this study. They were housed in solid-concrete-floor indoor pens (1.42 m × 3.86 m). Pigs were randomly assigned to 4 treatments: NC (negative control without AF and DON, 8 pens); PC (contaminated diet with AF at 150 µg/kg and DON at 1100 µg/kg, 8 pens); HT (PC + humate, 0.5%, 8 pens); and EM (PC with β-mannanase at 800 U/kg, 8 pens). There were 3 pigs per pen. The pigs had free access to feed and water throughout the duration of the experiment. Experimental diets (Table 10) were formulated to meet or exceed the nutrient requirements suggested by NRC [61]. The ADG, ADFI, and G:F ratio were calculated using measurements of feed intake and body weight taken weekly for 42 days.

### 4.4. Blood Sampling

Blood samples were collected aseptically from the jugular vein for all pigs on days 28 and 42. Blood was collected in Monovette tubes (Sarstedt, Newton, NC, USA) containing EDTA for hematological analysis. Tubes without anticoagulant were used to collect serum for measuring antibody titter, liver biochemistry, immunoglobulin, and cytokine concentrations. Serum samples were allowed to clot overnight at 4 °C before centrifuging for 15 min at 3000× *g* (4 °C), and were finally stored at −80 °C until analyzed.

### 4.5. Immune Parameters

Serum TNF-α was measured by ELISA following the instructions of a Porcine TNF-α DuoSet ELISA Kit (#DY690B, R&D Systems, Minneapolis, MN, USA) as previously described by a previous study [62]. A total of 50 µL assay diluent RD1-63 was added to microplate wells coated with a monoclonal antibody specific to porcine TNF-α, followed by 50 µL of standard, control, or sample. Detection occurred using a color reagent substrate and a stop solution of diluted hydrochloric acid. Absorbance was read at 450 nm and 540 nm by an ELISA plate reader (Synergy HT, BioTek Instruments, Winooski, VT, USA) and KC4 data analysis software.

Total concentration of the IgG was measured via ELISA, following instructions of the pig ELISA kit (#E101-104, Bethyl Laboratories, Montgomery, TX, USA) as described by a previous study [58]. Goat anti-pig IgG was used as the capture antibody to coat wells. Serum samples were diluted to 1:140,000. Horseradish peroxidase-labeled goat anti-pig IgG was used as the detection antibody in combination with the TMB (3,3′,5,5′-tetramethylbenzidene) enzyme substrate. A stop solution of 0.18 M sulfuric acid (H_2_SO_4_) was used to stop the enzyme-substrate reaction. Absorbance was read at 450 nm using an ELISA plate reader (Synergy HT, BioTek Instruments, Winooski, VT, USA) and KC4 data analysis software. Samples were quantified relative to a standard curve constructed with known amounts of pig immunoglobulin subset.

### 4.6. Hematological Measurements

Whole blood samples treated with EDTA were sent to Antech Diagnostics (Cary, NC, USA) for complete blood count on day 28 and day 42. Measurements included hematocrit, hemoglobin, mean corpuscular hemoglobin (MCH), mean corpuscular hemoglobin concentration (MCHC), mean corpuscular volume (MCV), platelet number, red blood cell count, white blood cell (WBC) count, basophils, eosinophils, lymphocytes, monocytes, and neutrophils.

### 4.7. Biochemical Serum Assays

Concentrations of alanine aminotransferase, albumin, alkaline phosphatase, aspartate aminotransferase, bilirubin, BUN-to-creatinine ratio (BUN:creatinine), calcium, chloride, cholesterol, creatinine, creatine phosphokinase (CPK), globulin, glucose, nitrogen, phosphorus, potassium, protein, sodium, and sodium-to-potassium ratio (Na:K) were measured (Antech Diagnostics, Cary, NC, USA) for determination of liver function on days 28 and 42.

### 4.8. Histological Measurements

On day 42, the median initial body weight pig from each pen was euthanized via captive bolt to collect liver, kidney, and spleen tissues for weight, color, and damage evaluation. Tissue color was measured from 3 locations on each tissue via a Minolta Colorimeter (Konica Minolta, Ramsey, NJ, USA), which measured values of lightness, redness, and yellowness. Samples from the liver and kidneys were fixed in 10% buffered formalin and sent to the North Carolina State University Histopathology Laboratory (College of Veterinary Medicine, Raleigh, NC, USA) for hematoxylin and eosin staining and observation of tissue damage. Liver damage measurement included bile duct hyperplasia, fibrosis, hydropic degeneration, inflammation, karyomegaly, necrosis, and vacuolation. Kidney damage measurement included fibrosis, inflammation, necrosis, protein casts, regeneration, and vacuolation. Microscopic examinations of tissue damage were measured by an evaluator blinded to treatment, based on the degree of change observed with values of 1: normal to minimal damage (0% to 5%); 2: mild (5% to 15%); 3: moderate (15% to 40%); 4: severe (higher than 40%) according to Weaver et al. [33].

### 4.9. Statistical Analysis

Data were analyzed using the MIXED procedures of SAS 9.4 software (SAS Inst., Inc., Cary, NC, USA) following a randomized complete block design. Dietary treatment was considered as a fixed effect and initial body weight was included as a random effect. A pen was considered as the experimental unit for growth performance. A pig was considered as the experimental unit for other response criteria. The least squares mean for each treatment was calculated. Preplanned contrasts were conducted to determine the impacts of AF and DON (NC vs. PC) and the effects of HT and EM on mitigating the impacts of AF and DON (PC vs. HT or EM). Probability values of less than 0.05 were considered statistically significant and those between 0.05 and 0.10 were considered as trends.

## Figures and Tables

**Table 1:** In vitro aflatoxin binding efficiency of humate, β-mannan hydrolysate, and commercially available mycotoxin-detoxifying additives.

Treatment	Adsorption, %	Desorption, %	Efficiency, %
Humate ^1^	28.5	6.1	22.4
β-mannan hydrolysate ^2^	22.2	5.6	16.6
Clay-based additive ^3^	92.0	0.8	91.2
Clay and yeast cell wall-based additive ^4^	75.2	5.7	69.5

^1^ Humate was obtained from Live Earth Product (Emory, UT, USA). ^2^ Guar gum (MP Biomedicals, LLC, Solon, OH, USA) was used as a source of β-mannans (40.0%) and digested by β-mannanase (CTCBIO, Seoul, Republic of Korea). ^3^ The clay-based additive (Calibrin^®^-A Enterosorbent) was composed of a processed calcium montmorillonite clay and obtained from a commercial company (Amlan International, Chicago, IL, USA). ^4^ The clay and yeast cell wall-based additive (Unike, Nutriad International NV, Sint-Niklaas, Belgium) was composed of sodium bentonite, sepiolite clay, and brewer’s dried yeast.

**Table 2:** Serum immune markers on day 28 and 42 in pigs fed experimental diets with or without mycotoxins and supplemented with humate (0.5%) or β-mannanase (800 U/kg).

Item	Treatment ^1^				SEM ^2^	*p*-Value		
	NC	PC	HT	EM		NC vs. PC	PC vs. HT	PC vs. EM
d 28								
TNF-α ^3^, mg/mL	150	116	124	116	9	0.011	0.546	0.999
IgG ^4^, mg/mL	1.26	1.06	1.06	1.29	0.12	0.264	0.976	0.201
d 42								
TNF-α, mg/mL	135	99.9	99.8	98.9	19	0.191	0.997	0.971
IgG, mg/mL	0.94	1.15	0.96	0.83	0.09	0.062	0.090	0.007

^1^ NC = uncontaminated diet; PC = contaminated diet with aflatoxin B_1_ at 150 µg/kg and 1100 deoxynivalenol at µg/kg; HT = PC diet with humate at 0.5%; EM = PC diet with β-mannanase at 800 U/kg. ^2^ SEM = standard error of the mean. Each least squares mean represents 8 observations, with a pig as the experimental unit. ^3^ TNF-α = tumor necrosis factor-α. ^4^ IgG = immunoglobulin G.

**Table 3:** Hematological profiles on day 28 in pigs fed diets with or without mycotoxins and supplemented with humate (0.5%) or β-mannanase (800 U/kg).

Item ^1^	Treatment ^2^				SEM ^3^	*p*-Value		
	NC	PC	HT	EM		NC vs. PC	PC vs. HT	PC vs. EM
Hematocrit, %	32.6	36.7	35.1	40.7	2.4	0.235	0.649	0.256
Hemoglobin, g/dL	10.5	11.9	11.3	12.9	0.7	0.168	0.526	0.362
MCH, pg	15.8	17.2	16.5	16.2	0.3	0.003	0.093	0.027
MCHC, g/dL	32.1	32.5	32.1	31.9	0.3	0.269	0.227	0.078
MCV, fL	49.3	52.7	51.3	50.8	1.0	0.022	0.334	0.196
Platelet, 10^3^/μL	368	427	407	419	77	0.498	0.813	0.927
RBC, 10^6^/μL	6.61	6.99	6.84	8.03	0.48	0.568	0.818	0.131
WBC, 10^3^/μL	27.1	25.5	28.6	22.8	2.0	0.577	0.293	0.364
Basophil, 10^3^/μL	0.29	0.16	0.19	0.20	0.04	0.018	0.497	0.440
Eosinophil, 10^3^/μL	0.60	0.56	0.86	0.46	0.17	0.871	0.198	0.684
Lymphocyte, 10^3^/μL	12.9	11.1	12.4	9.7	1.0	0.127	0.256	0.247
Monocyte, 10^3^/μL	1.97	2.10	2.02	1.73	0.22	0.686	0.810	0.246
Neutrophil, 10^3^/μL	10.9	11.0	12.4	10.3	1.5	0.936	0.527	0.740

^1^ MCH = mean corpuscular hemoglobulin; MCHC = mean corpuscular hemoglobulin concentration; MCV = mean corpuscular volume; RBC = red blood cell; WBC = white blood cell. ^2^ NC = uncontaminated diet; PC = contaminated diet with aflatoxin B_1_ at 150 µg/kg and 1100 deoxynivalenol at µg/kg; HT = PC diet with humate at 0.5%; EM = PC diet with β-mannanase at 800 U/kg. ^3^ SEM = standard error of the mean. Each least squares mean represents 8 observations, with a pig as the experimental unit.

**Table 4:** Hematological profiles on day 42 in pigs fed diets with or without mycotoxins and supplemented with humate (0.5%) or β-mannanase (800 U/kg).

Item ^1^	Treatment ^2^				SEM ^3^	*p*-Value		
	NC	PC	HT	EM		NC vs. PC	PC vs. HT	PC vs. EM
Hematocrit, %	34.8	37.4	36.2	37.1	1.28	0.080	0.414	0.832
Hemoglobin, g/dL	11.0	11.4	11.6	11.8	0.36	0.387	0.644	0.418
MCH, pg	17.2	18.0	17.4	17	0.27	0.037	0.121	0.021
MCHC, g/dL	31.6	30.5	32.0	31.8	0.56	0.107	0.016	0.068
MCV, fL	54.4	59.1	53.9	54.0	0.9	<0.001	<0.001	<0.001
Platelet, 10^3^/μL	452	416	340	384	33	0.433	0.120	0.487
RBC, 10^6^/μL	6.39	6.33	6.70	6.88	0.20	0.825	0.204	0.052
WBC, 10^3^/μL	19.0	21.6	20.2	18.3	1.8	0.312	0.601	0.194
Basophil, 10^3^/μL	0.06	0.19	0.12	0.14	0.03	0.008	0.159	0.240
Eosinophil, 10^3^/μL	0.62	0.74	0.51	0.34	0.14	0.530	0.267	0.052
Lymphocyte, 10^3^/μL	9.56	9.25	8.67	9.95	0.77	0.717	0.504	0.406
Monocyte, 10^3^/μL	1.08	1.45	1.04	1.04	0.18	0.074	0.059	0.047
Neutrophil, 10^3^/μL	7.72	9.95	9.99	6.83	1.48	0.289	0.985	0.142

^1^ MCH = mean corpuscular hemoglobulin; MCHC = mean corpuscular hemoglobulin concentration; MCV = mean corpuscular volume; RBC = red blood cell; WBC = white blood cell. ^2^ NC = uncontaminated diet; PC = contaminated diet with aflatoxin B1 at 150 µg/kg and 1100 deoxynivalenol at µg/kg; HT = PC diet with mined humate at 0.5%; EM = PC diet with β-mannanase at 800 U/kg. ^3^ SEM = standard error of the mean. Each least squares mean represents 8 observations, with a pig as the experimental unit.

**Table 5:** Serum biochemistry on day 28 in pigs fed diets with or without mycotoxins and supplemented with humate (0.5%) or β-mannanase (800 U/kg).

Item ^1^	Treatment ^2^				SEM ^3^	*p*-Value		
	NC	PC	HT	EM		NC vs. PC	PC vs. HT	PC vs. EM
Glucose, mg/dL	92.4	85.1	90.5	100.6	8.6	0.500	0.589	0.129
Total protein, g dL	5.80	5.84	6.05	6.24	0.24	0.915	0.514	0.224
BUN, mg/dL	13.1	15.3	15.0	16.3	1.0	0.109	0.833	0.401
CRT, mg/dL	0.67	0.60	0.63	0.69	0.03	0.053	0.446	0.012
BUN:CRT	19.6	25.5	24.3	23.8	1.7	0.002	0.430	0.273
ALB, g/dL	2.03	2.23	2.48	2.34	0.10	0.206	0.079	0.418
GLOB, g/dL	3.77	3.61	3.58	3.90	0.28	0.709	0.922	0.453
ALB:GLOB	0.58	0.63	0.70	0.65	0.06	0.638	0.363	0.760
AST, U/L	39.5	47.6	41.6	39.6	4.7	0.248	0.351	0.217
ALT, U/L	31.0	29.8	31.6	33.1	5.1	0.847	0.762	0.586
Cholesterol, mg/dL	71.7	83.8	80.3	80.6	5.7	0.160	0.655	0.689
Sodium, mEq/L	140	142	142	143	1.0	0.170	0.803	0.561
Potassium, mEq/L	6.14	6.00	6.40	6.68	0.39	0.764	0.351	0.122
Na:K	23.3	24.3	22.9	21.6	1.3	0.521	0.338	0.075
Chloride, mEq/L	99.3	98.4	98.8	99.0	1.0	0.522	0.786	0.652
Calcium, mg/dL	10.1	10.2	10.1	10.2	0.2	0.760	0.845	0.938
Phosphorus, mg/dL	8.68	9.76	9.70	9.63	0.4	0.059	0.902	0.786
Alkaline phosphatase, U/L	241	253	178	227	35	0.801	0.093	0.551
CPK, U/L	698	304	595	292	140	0.067	0.139	0.947
Bilirubin, g/dL	0.100	0.113	0.100	0.100	0.007	0.218	0.185	0.185

^1^ BUN = blood urea nitrogen; CRT = creatinine; BUN:CRT = BUN to CRT ratio; ALB = albumin; GLOB = globulin; ALB:GLOB = ALB-to-GLOB ratio; AST = aspartate aminotransferase; ALT= alanine aminotransferase; Na:K = sodium-to-potassium ratio; CPK = creatine phosphokinase. ^2^ NC = uncontaminated diet; PC = contaminated diet with aflatoxin B_1_ at 150 µg/kg and 1100 deoxynivalenol at µg/kg; HT = PC diet with humate at 0.5%; EM = PC diet with β-mannanase at 800 U/kg. ^3^ SEM = standard error of the mean. Each least squares mean represents 8 observations, with a pig as the experimental unit.

**Table 6:** Serum biochemistry on day 42 in pigs fed diets with or without mycotoxins and supplemented with humate (0.5%) or β-mannanase (800 U/kg).

Item ^1^	Treatment ^2^				SEM ^3^	*p*-Value		
	NC	PC	HT	EM		NC vs. PC	PC vs. HT	PC vs. EM
Glucose, mg/dL	112	111	111	115	5	0.834	0.983	0.505
Total protein, g dL	5.66	5.98	5.98	5.83	0.18	0.222	1.000	0.553
BUN, mg/dL	13.0	13.0	12.9	13.1	0.6	1.000	0.874	0.874
CRT, mg/dL	0.66	0.65	0.64	0.61	0.03	0.792	0.792	0.431
BUN:CRT	20.0	20.1	20.6	22.0	1.1	0.936	0.747	0.232
ALB, g/dL	2.38	2.66	2.74	2.94	0.15	0.159	0.708	0.177
GLOB, g/dL	3.29	3.31	3.24	2.89	0.22	0.938	0.815	0.190
ALB:GLOB	0.78	0.85	0.90	1.04	0.09	0.554	0.693	0.145
AST, U/L	31.3	46.4	45.1	39.4	4.6	0.026	0.847	0.286
ALT, U/L	28.6	28.9	35.9	32.3	2.7	0.947	0.073	0.376
Cholesterol, mg/dL	77.8	78.4	75.6	73.1	3.6	0.902	0.590	0.306
Sodium, mEq/L	143	143	143	143	1	0.774	0.924	1.000
Potassium, mEq/L	5.54	5.75	5.49	5.75	0.23	0.497	0.403	1.000
Na:K	26.0	25.1	26.5	25.1	1.2	0.567	0.371	1.000
Chloride, mEq/L	100	100	101	102	1	0.798	0.552	0.133
Calcium, mg/dL	9.83	10.31	10.11	10.15	0.18	0.042	0.387	0.481
Phosphorus, mg/dL	8.88	9.8	9.61	9.44	0.31	0.023	0.628	0.351
Alkaline phosphatase, U/L	229	242	256	240	24	0.714	0.674	0.962
CPK, U/L	462	954	925	531	292	0.244	0.945	0.315

^1^ BUN = blood urea nitrogen; CRT = creatinine; BUN:CRT = BUN-to-CRT ratio; ALB = albumin; GLOB = globulin; ALB:GLOB = ALB-to-GLOB ratio; AST = aspartate aminotransferase; ALT= alanine aminotransferase; Na:K = sodium-to-potassium ratio; CPK = creatine phosphokinase. ^2^ NC = uncontaminated diet; PC = contaminated diet with aflatoxin B_1_ at 150 µg/kg and 1100 deoxynivalenol at µg/kg; HT = PC diet with humate at 0.5%; EM = PC diet with β-mannanase at 800 U/kg. ^3^ SEM = standard error of the mean. Each least squares mean represents 8 observations, with a pig as the experimental unit. Serum bilirubin concentrations below the detection limit (0.1 g/dL) were recorded as 0.1 g/dL; thus, all samples showed the same value and were excluded from the statistical analysis.

**Table 7:** Organ weight and organ color of pigs fed diets with or without mycotoxins and supplemented with humate (0.5%) or β-mannanase (800 U/kg).

Item	Treatment ^1^				SEM ^2^	*p*-Value		
	NC	PC	HT	EM		NC vs. PC	PC vs. HT	PC vs. EM
Organ weight, g								
Liver	692	776	718	765	39	0.142	0.307	0.851
Kidney	136	138	134	134	9	0.831	0.731	0.725
Spleen	59.4	49.8	55.0	55.3	4.9	0.047	0.264	0.243
Relative organ weight ^3^, %								
Liver	2.70	3.25	2.91	3.08	0.15	0.004	0.052	0.304
Kidney	0.528	0.580	0.542	0.537	0.029	0.208	0.350	0.295
Spleen	0.230	0.208	0.221	0.222	0.013	0.219	0.475	0.450
Color ^4^								
Liver								
L	34.8	36.2	35.1	36.6	0.8	0.219	0.322	0.686
a	13.9	13.9	13.8	14.3	0.3	0.958	0.649	0.274
b	2.96	3.77	4.09	4.58	0.45	0.216	0.619	0.215
Kidney								
L	46.4	46.2	45.1	46.9	1.0	0.870	0.448	0.620
a	12.5	13.1	13.6	11.8	0.7	0.578	0.581	0.216
b	8.07	7.05	8.68	8.36	0.67	0.292	0.097	0.178
Spleen								
L	34.6	36.3	37.0	37.5	0.6	0.072	0.417	0.165
a	17.7	18.6	18.6	18.9	0.3	0.037	0.890	0.491
b	1.69	1.92	2.75	1.77	0.45	0.721	0.209	0.813

^1^ NC = uncontaminated diet; PC = contaminated diet with aflatoxin B_1_ at 150 µg/kg and 1100 deoxynivalenol at µg/kg; HT = PC diet with humate at 0.5%; EM = PC diet with β-mannanase at 800 U/kg. ^2^ SEM = standard error of the mean. ^3^ Relative organ weight (%) was calculated as organ weight (g) ÷ body weight (kg) ÷ 1000 × 100. ^4^ Tissue color was measured using a Minolta colorimeter (Konica Minolta, Ramsey, NJ, USA): L = lightness; a = redness; b = yellowness. Each least squares mean represents 8 observations, with a pig as the experimental unit.

**Table 8:** Liver and kidney tissue damage in pigs fed diets with or without mycotoxins and supplemented with humate (0.5%) or β-mannanase (800 U/kg).

Item	Treatment ^1^				SEM ^2^	*p*-Value		
	NC	PC	HT	EM		NC vs. PC	PC vs. HT	PC vs. EM
Liver tissue damage ^3^, %								
Bile duct hyperplasia	2.63	4.75	3.00	3.00	0.61	0.019	0.049	0.049
Fibrosis	4.75	3.00	3.00	3.00	0.57	0.040	1.000	1.000
Hydropic degeneration	9.10	10.0	8.30	10.00	0.7	0.398	0.097	1.000
Inflammation	2.25	3.00	2.63	2.63	0.36	0.153	0.469	0.469
Karyomegaly	2.6	30.0	22.1	28.9	8.5	0.004	0.365	0.896
Necrosis	2.63	2.63	2.63	3.00	0.33	1.000	1.000	0.416
Vacuolation	3.88	6.50	3.88	8.25	1.10	0.089	0.089	0.250
Kidney tissue damage ^3^, %								
Fibrosis	2.63	3.00	2.25	2.63	0.37	0.458	0.144	0.458
Necrosis	3.00	2.63	2.63	2.63	0.32	0.421	1.000	1.000
Protein casts	2.63	2.63	2.63	2.25	0.44	1.000	1.000	0.497
Regeneration	2.25	3.00	2.63	2.63	0.36	0.153	0.469	0.469
Vacuolation	2.63	3.88	3.88	4.75	0.86	0.315	1.000	0.480

^1^ NC = uncontaminated diet; PC = contaminated diet with aflatoxin B_1_ at 150 µg/kg and 1100 deoxynivalenol at µg/kg; HT = PC diet with humate at 0.5%; EM = PC diet with β-mannanase at 800 U/kg. ^2^ SEM = standard error of the mean. ^3^ Tissue damage (%) was determined as the percentage of damaged tissue relative to the total tissue area. Measured percentages can be categorized into the following groups: normal to minimal (<5%); mild (5 to 15%); moderate (15 to 40%); severe (>40%). Each least squares mean represents 8 observations, with a pig as the experimental unit.

**Table 9:** Growth performance in pigs fed diets with or without mycotoxins and supplemented with humate (0.5%) or β-mannanase (800 U/kg).

Item	Treatment ^1^				SEM ^2^	*p*-Value		
	NC	PC	HT	EM		NC vs. PC	PC vs. HT	PC vs. EM
Body weight, kg								
d 0	8.7	8.7	8.5	8.9	0.5	0.691	0.462	0.254
d 7	9.1	9.4	8.9	9.7	0.8	0.521	0.355	0.624
d 14	10.8	11.3	10.3	11.5	0.9	0.589	0.225	0.720
d 21	13.3	13.6	12.4	13.6	1.0	0.719	0.168	0.960
d 28	16.8	16.2	17.0	17.4	1.3	0.541	0.459	0.260
d 35	20.6	19.2	20.6	21.0	1.5	0.271	0.262	0.157
d 42	25.7	23.9	24.9	25.2	1.7	0.160	0.435	0.298
ADG, g/d								
d 0 to 7	50	108	58	114	54	0.384	0.453	0.925
d 7 to 14	249	263	192	268	35	0.780	0.161	0.922
d 14 to 21	352	335	301	301	49	0.778	0.568	0.569
d 21 to 28	505	372	655	531	81	0.252	0.020	0.174
d 28 to 35	533	432	518	513	54	0.105	0.167	0.192
d 35 to 42	736	663	613	610	41	0.102	0.260	0.231
Overall	404	362	389	389	30	0.143	0.339	0.338
ADFI, g/d								
d 0 to 7	289	286	242	324	49	0.937	0.328	0.401
d 7 to 14	481	530	452	573	56	0.467	0.252	0.520
d 14 to 21	681	608	603	629	55	0.294	0.938	0.762
d 21 to 28	847	830	871	843	73	0.844	0.640	0.881
d 28 to 35	952	892	1032	951	86	0.516	0.138	0.528
d 35 to 42	1314	1268	1326	1213	87	0.661	0.584	0.605
Overall	761	736	754	755	56	0.655	0.744	0.728
G:F ^3^								
d 0 to 7	0.09	0.38	–0.07	0.24	0.17	0.186	0.044	0.498
d 7 to 14	0.50	0.50	0.41	0.46	0.05	0.942	0.225	0.660
d 14 to 21	0.53	0.55	0.52	0.47	0.07	0.860	0.750	0.435
d 21 to 28	0.68	0.45	0.73	0.62	0.11	0.150	0.077	0.286
d 28 to 35	0.58	0.48	0.50	0.54	0.04	0.066	0.698	0.252
d 35 to 42	0.57	0.53	0.47	0.51	0.03	0.285	0.176	0.644
Overall	0.54	0.49	0.51	0.52	0.02	0.037	0.397	0.293

^1^ NC = uncontaminated diet; PC = contaminated diet with aflatoxin B_1_ at 150 µg/kg and 1100 deoxynivalenol at µg/kg; HT = PC diet with humate at 0.5%; EM = PC diet with β-mannanase at 800 U/kg. ^2^ SEM = standard error of the mean. ^3^ G:F = gain-to-feed ratio. Each least squares mean represents 8 observations, with a pen as the experimental unit. The number of pigs per pen was 3 for NC and PC treatments and 2 for HT and EM treatments.

**Table 10:** Composition of experimental diets ^1^.

Item	Treatment	
	NC	PC
Ingredient, %		
Ground corn	69.42	4.42
Ground barley	3.00	0.00
Ground corn with aflatoxin ^2^	0.00	65.00
Ground barley with deoxynivalenol ^3^	0.00	3.00
Soybean meal, dehulled	25.00	25.00
Poultry fat	0.50	0.50
Dicalcium phosphate	0.90	0.90
Ground limestone	0.70	0.70
Salt	0.30	0.30
Vitamin premix ^4^	0.03	0.03
Trace mineral premix ^5^	0.15	0.15
Calculated composition		
Dry matter, %	89.50	89.50
ME, Mcal/kg	3.36	3.36
Crude protein, %	18.08	18.08
Lys, %	0.95	0.95
Met + Cys, %	0.61	0.61
Trp, %	0.21	0.21
Thr, %	0.68	0.68
Calcium, %	0.61	0.61
Total phosphorus, %	0.54	0.54
Available phosphorus, %	0.23	0.23
Aflatoxin B_1_, µg/kg	0.00	175
Deoxynivalenol, µg/kg	0.00	900
Analyzed composition		
Dry matter, %	88.55	89.97
Crude protein, %	16.71	19.34
Aflatoxin ^6^, µg/kg	<20	150
Deoxynivalenol ^6^, µg/kg	<500	1100
Fumonisin ^6^, µg/kg	<2000	3000
Zearalenone ^6^, µg/kg	<500	<500

^1^ Humate (0.5% of the complete diet) or β-mannanase (0.1% of the complete diet; 800 U/kg feed, CTCBIO Inc., Seoul, Republic of Korea) were supplemented at the expense of ground corn (uncontaminated) in PC diet to formulate the HT or EM diet. Analyzed activity of β-mannanase in the EN diet was 986 U/kg feed. ^2^ Corn contained 270 µg/kg aflatoxin. ^3^ Barley contained 30,000 µg/kg deoxynivalenol. ^4^ The vitamin premix provided the following per kilogram of complete diet: 6613.8 IU of vitamin A as vitamin A acetate; 992.0 IU of vitamin D_3_; 19.8 IU of vitamin E; 2.64 mg of vitamin K as menadione sodium bisulfate; 0.03 mg of vitamin B_12_; 4.63 mg of riboflavin; 18.52 mg of D-pantothenic acid as calcium pantothenate; 24.96 mg of niacin; and 0.07 mg of biotin. ^5^ The trace mineral premix provided the following per kilogram of complete diet: 4.0 mg of Mn as manganous oxide; 165 mg of Fe as ferrous sulfate; 165 mg of Zn as zinc sulfate; 16.5 mg of Cu as copper sulfate; 0.30 mg of I as ethylenediamine dihydroiodide; and 0.30 mg of Se as sodium selenite. ^6^ Mycotoxin concentration based on average obtained from analysis by North Dakota State University Veterinary Diagnostic Laboratory (Fargo, ND, USA).

## Data Availability

The original contributions presented in this study are included in the article. Further inquiries can be directed to the corresponding author.

## References

[1] Chaytor A.C., Hansen J.A., van Heugten E., See M.T., Kim S.W. (2011). Occurrence and decontamination of mycotoxins in swine feed. Asian-Australas. J. Anim. Sci..

[2] Choi H., Garavito-Duarte Y., Gormley A.R., Kim S.W. (2025). Aflatoxin B_1_: Challenges and strategies for the intestinal microbiota and intestinal health of monogastric animals. Toxins.

[3] Patriarca A., Fernández Pinto V. (2017). Prevalence of mycotoxins in foods and decontamination. Curr. Opin. Food Sci..

[4] Gruber-Dorninger C., Jenkins T., Schatzmayr G. (2019). Global mycotoxin occurrence in feed: A ten-year survey. Toxins.

[5] Holanda D.M., Kim Y.I., Parnsen W., Kim S.W. (2021). Phytobiotics with adsorbent to mitigate toxicity of multiple mycotoxins on health and growth of pigs. Toxins.

[6] Holanda D.M., Yiannikouris A., Kim S.W. (2020). Investigation of the efficacy of a postbiotic yeast cell wall-based blend on newly-weaned pigs under a dietary challenge of multiple mycotoxins with emphasis on deoxynivalenol. Toxins.

[7] Holanda D.M., Kim S.W. (2021). Mycotoxin occurrence, toxicity, and detoxifying agents in pig production with an emphasis on deoxynivalenol. Toxins.

[8] Chlebicz A., Śliżewska K. (2020). In vitro detoxification of aflatoxin B_1_, deoxynivalenol, fumonisins, T-2 toxin and zearalenone by probiotic bacteria from genus *Lactobacillus* and *Sacharomyces cerevisiae* yeast. Probiotics Antimicrob. Proteins.

[9] Park S.-H., Kim J., Kim D., Moon Y. (2017). Mycotoxin detoxifiers attenuate deoxynivalenol-induced pro-inflammatory barrier insult in porcine enterocytes as an in vitro evaluation model of feed mycotoxin reduction. Toxicol. In Vitro.

[10] Xiong D., Wen J., Lu G., Li T., Long M. (2022). Isolation, purification, and characterization of a laccase-degrading aflatoxin B1 from *Bacillus amyloliquefaciens* B10. Toxins.

[11] Holanda D.M., Kim S.W. (2020). Efficacy of mycotoxin detoxifiers on health and growth of newly-weaned pigs under chronic dietary challenge of deoxynivalenol. Toxins.

[12] Aiken G.R., McKnight D.M., Wershaw R.L., MacCarthy P. (1986). Humic Substances in Soil, Sediment, and Water: Geochemistry, Isolation and Characterization.

[13] Ji F., McGlone J.J., Kim S.W. (2006). Effects of dietary humic substances on pig growth performance, carcass characteristics, and ammonia emission. J. Anim. Sci..

[14] Maffia A., Oliva M., Marra F., Mallamaci C., Nardi S., Muscolo A. (2025). Humic substances: Bridging ecology and agriculture for a greener future. Agronomy.

[15] Janoš P., Kormunda M., Novák F., Životský O., Fuitová J., Pilařová V. (2013). Multifunctional humate-based magnetic sorbent: Preparation, properties and sorption of Cu (II), phosphates and selected pesticides. React. Funct. Polym..

[16] van Rensburg C.J., Van Rensburg C.E.J., Van Ryssen J.B.J., Casey N.H., Rottinghaus G.E. (2006). In vitro and in vivo assessment of humic acid as an aflatoxin binder in broiler chickens. Poult. Sci..

[17] Arafat R.Y., Khan S.H., Saima N. (2017). Evaluation of humic acid as an aflatoxin binder in broiler chickens. Ann. Anim. Sci..

[18] Maguey-González J.A., Nava-Ramírez M.d.J., Gómez-Rosales S., Ángeles M.d.L., Solís-Cruz B., Hernández-Patlán D., Merino-Guzmán R., Hernandez-Velasco X., Hernández-Ramírez J.O., Loeza I. (2023). Evaluation of the efficacy of humic acids to counteract the toxic effects of aflatoxin B_1_ in turkey poults. Front. Vet. Sci..

[19] Jang K.B., Zhao Y., Kim Y.I., Pasquetti T., Kim S.W. (2023). Effects of bacterial β-mannanase on apparent total tract digestibility of nutrients in various feedstuffs fed to growing pigs. Anim. Biosci..

[20] Jang J.-C., Kim K.H., Jang Y.D., Kim Y.Y. (2020). Effects of dietary β-mannanase supplementation on growth performance, apparent total tract digestibility, intestinal integrity, and immune responses in weaning pigs. Animals.

[21] Jang K.B., Kim Y.I., Duarte M.E., Kim S.W. (2024). Effects of β-mannanase supplementation on intestinal health and growth of nursery pigs. J. Anim. Sci..

[22] Jahanian E., Mahdavi A.H., Asgary S., Jahanian R. (2016). Effect of dietary supplementation of mannanoligosaccharides on growth performance, ileal microbial counts, and jejunal morphology in broiler chicks exposed to aflatoxins. Livest. Sci..

[23] Zaghini A., Martelli G., Roncada P., Simioli M., Rizzi L. (2005). Mannanoligosaccharides and aflatoxin B_1_ in feed for laying hens: Effects on egg quality, aflatoxins B_1_ and M_1_ residues in eggs, and aflatoxin B_1_ levels in liver. Poult. Sci..

[24] Wu Y., Ren A., Lv X., Ran T., Zhang G., Zhou C., Tan Z. (2022). Effects of galactomannan oligosaccharides on growth performance, mycotoxin detoxification, serum biochemistry, and hematology of goats fed mycotoxins-contaminated diets. Front. Vet. Sci..

[25] Tiwari U.P., Chen H., Kim S.W., Jha R. (2018). Supplemental effect of xylanase and mannanase on nutrient digestibility and gut health of nursery pigs studied using both in vivo and in vitro models. Anim. Feed Sci. Technol..

[26] Marin D.E., Taranu I., Bunaciu R.P., Pascale F., Tudor D.S., Avram N., Sarca M., Cureu I., Criste R.D., Suta V. (2002). Changes in performance, blood parameters, humoral and cellular immune responses in weanling piglets exposed to low doses of aflatoxin. J. Anim. Sci..

[27] Ensley S.M., Radke S.L., Zimmerman J.J., Karriker L.A., Ramirez A., Schwartz K.J., Stevenson G.W., Zhang J. (2019). Mycotoxins in grains and feeds. Diseases of Swine.

[28] Mehrzad J., Devriendt B., Baert K., Cox E. (2015). Aflatoxins of type B and G affect porcine dendritic cell maturation in vitro. J. Immunotoxicol..

[29] Park K.M., Bowers W.J. (2010). Tumor necrosis factor-alpha mediated signaling in neuronal homeostasis and dysfunction. Cell Signal..

[30] Kawai T., Akira S. (2007). Signaling to NF-κB by Toll-like receptors. Trends Mol. Med..

[31] Liu D., Wang Q., He W., Chen X., Wei Z., Huang K. (2020). Two-way immune effects of deoxynivalenol in weaned piglets and porcine alveolar macrophages: Due mainly to its exposure dosage. Chemosphere.

[32] Bondy G.S., Pestka J.J. (2000). Immunomodulation by fungal toxins. J. Toxicol. Environ. Health Part B Crit. Rev..

[33] Weaver A.C., See M.T., Hansen J.A., Kim Y.B., De Souza A.L.P., Middleton T.F., Kim S.W. (2013). The use of feed additives to reduce the effects of aflatoxin and deoxynivalenol on pig growth, organ health and immune status during chronic exposure. Toxins.

[34] Deng Z., Jang K.B., Jalukar S., Du X., Kim S.W. (2023). Efficacy of feed additive containing bentonite and enzymatically hydrolyzed yeast on intestinal health and growth of newly weaned pigs under chronic dietary challenges of fumonisin and aflatoxin. Toxins.

[35] Jia R., Liu W., Zhao L., Cao L., Shen Z. (2020). Low doses of individual and combined deoxynivalenol and zearalenone in naturally moldy diets impair intestinal functions via inducing inflammation and disrupting epithelial barrier in the intestine of piglets. Toxicol. Lett..

[36] Pierron A., Alassane-Kpembi I., Oswald I.P. (2016). Impact of mycotoxin on immune response and consequences for pig health. Anim. Nutr..

[37] Nordgreen J., Edwards S.A., Boyle L.A., Bolhuis J.E., Veit C., Sayyari A., Marin D.E., Dimitrov I., Janczak A.M., Valros A. (2020). A proposed role for pro-inflammatory cytokines in damaging behavior in pigs. Front. Vet. Sci..

[38] Khatoon A., Zargham K.M., Abidin Z.U., Bhatti S.A. (2018). Effects of feeding bentonite clay upon ochratoxin A–induced immunosuppression in broiler chicks. Food Addit. Contam. Part A-Chem..

[39] Roque B.M., Reyes G.C., Tewoldebrhan T.A., Apphuamy J.A.D.R.N., Lee J.J., Seo S., Kebreab E. (2019). Exogenous β-mannanase supplementation improved immunological and metabolic responses in lactating dairy cows. J. Dairy Sci..

[40] Kiarie E.G., Steelman S., Martinez M. (2022). Does supplementing β-mannanase modulate the feed-induced immune response and gastrointestinal ecology in poultry and pigs? An appraisal. Front. Anim. Sci..

[41] Gomez-Rosales S., Angeles M.d.L., Tellez-Isaias G., Makan A. (2022). Mechanisms of action of humic substances as growth promoters in animals. Humus and Humic Substances—Recent Advances.

[42] Li D., Zhang Q., Ruan Z., Zhang Y., Liu X., Zhang G., Zhao H., Li J., Wu B. (2024). The relationship between mean corpuscular hemoglobin concentration and mortality in hypertensive individuals: A population-based cohort study. PLoS ONE.

[43] Doig K., Zhang B. (2017). A methodical approach to interpreting the red blood cell parameters of the complete blood count. Am. Soc. Clin. Lab. Sci..

[44] Veda P. (2013). Evaluation of macrocytosis in routine hemograms. Indian J. Hematol. Blood Transfus..

[45] Shen S., Yan X., Xu B. (2022). The blood urea nitrogen/creatinine (BUN/cre) ratio was U-shaped associated with all-cause mortality in general population. Ren. Fail..

[46] Moissl A.P., Delgado G.E., Kleber M.E., Krämer B.K., März W., Lorkowski S. (2024). Associations between serum mineral concentrations and mortality by renal function in the Ludwigshafen Risk and cardiovascular health study. Sci. Rep..

[47] Tessari E.N., Kobashigawa E., Cardoso A.L., Ledoux D.R., Rottinghaus G.E., Oliveira C.A. (2010). Effects of aflatoxin B(1) and fumonisin B(1) on blood biochemical parameters in broilers. Toxins.

[48] Gbore F., Egbunike G. (2009). Toxicological evaluation of dietary fumonisin B_1_ on serum biochemistry of growing pigs. J. Cent. Eur. Agric..

[49] Cheng Y.-H., Weng C.-F., Chen B.-J., Chang M.-H. (2006). Toxicity of different Fusarium mycotoxins on growth performance, immune responses and efficacy of a mycotoxin degrading enzyme in pigs. Anim. Res..

[50] Andretta I., Kipper M., Lehnen C.R., Hauschild L., Vale M.M., Lovatto P.A. (2012). Meta-analytical study of productive and nutritional interactions of mycotoxins in growing pigs. Animal.

[51] Liu X., Kumar Mishra S., Wang T., Xu Z., Zhao X., Wang Y., Yin H., Fan X., Zeng B., Yang M. (2020). AFB_1_ induced transcriptional regulation related to apoptosis and lipid metabolism in liver of chicken. Toxins.

[52] Kuang Y., Wu Z., Liu Y. (2025). Deoxynivalenol induces spleen damage, apoptosis, and inflammation in mice by increasing mitochondrial reactive oxygen species: Protective effects of curcumin. Food Chem. Toxicol..

[53] Li P., Su R., Yin R., Lai D., Wang M., Liu Y., Zhou L. (2020). Detoxification of mycotoxins through biotransformation. Toxins.

[54] Domijan A., Zeljezic D., Peraica M., Kovacevic G., Gregorovic G., Krstanac Z., Horvatin K., Kalafatic M. (2008). Early toxic effects of fumonisin B_1_ in rat liver. Hum. Exp. Toxicol..

[55] Hard G.C. (2018). Critical review of renal tubule karyomegaly in non-clinical safety evaluation studies and its significance for human risk assessment. Crit. Rev. Toxicol..

[56] Holanda D.M., Kim S.W. (2021). Investigation of the efficacy of mycotoxin-detoxifying additive on health and growth of newly-weaned pigs under deoxynivalenol challenges. Anim. Biosci..

[57] Weaver A.C., See M.T., Kim S.W. (2014). Protective effect of two yeast based feed additives on pigs chronically exposed to deoxynivalenol and zearalenone. Toxins.

[58] Kim S.W., Holanda D.M., Gao X., Park I., Yiannikouris A. (2019). Efficacy of a yeast cell wall extract to mitigate the effect of naturally co-occurring mycotoxins contaminating feed ingredients fed to young pigs: Impact on gut health, microbiome, and growth. Toxins.

[59] Ripken D., van der Wielen N., Wortelboer H.M., Meijerink J., Witkamp R.F., Hendriks H.F.J. (2016). Nutrient-induced glucagon like peptide-1 release is modulated by serotonin. J. Nutr. Biochem..

[60] Pasternak J.A., Aiyer V.I.A., Hamonic G., Beaulieu A.D., Columbus D.A., Wilson H.L. (2018). Molecular and physiological effects on the small intestine of weaner pigs following feeding with deoxynivalenol-contaminated feed. Toxins.

[61] NRC (2012). Nutrient Requirements of Swine.

[62] Cheng Y.C., Duarte M.E., Kim S.W. (2022). Effects of *Yarrowia lipolytica* supplementation on growth performance, intestinal health and apparent ileal digestibility of diets fed to nursery pigs. Anim. Biosci..

